# The impact of national cancer awareness campaigns for bowel and lung cancer symptoms on sociodemographic inequalities in immediate key symptom awareness and GP attendances

**DOI:** 10.1038/bjc.2015.31

**Published:** 2015-03-03

**Authors:** J Moffat, A Bentley, L Ironmonger, A Boughey, G Radford, S Duffy

**Affiliations:** 1 Cancer Research UK, Angel Building, 407 St John Street, London EC1V 4AD, UK; 2 Anglia and Essex Centre Director, Public Health England, Eastbrook, Shaftesbury Road, Cambridge CB2 8DF, UK; 3 National Health Service England, Quarry House, Quarry Hill, Leeds LS2 7UE, UK

**Keywords:** lung cancer, bowel cancer, colorectal cancer, awareness, GP attendance, health campaign, inequalities, men

## Abstract

**Background:**

National campaigns focusing on key symptoms of bowel and lung cancer ran in England in 2012, targeting men and women over the age of 50 years, from lower socioeconomic groups.

**Methods:**

Data from awareness surveys undertaken with samples of the target audience (*n*=1245/1140 pre-/post-bowel campaign and *n*=1412/1246 pre-/post-lung campaign) and Read-code data extracted from a selection general practitioner (GP) practices (*n*=355 for bowel and *n*=486 for lung) were analysed by population subgroups.

**Results:**

*Unprompted symptom awareness:* There were no significant differences in the magnitude of shift in ABC1 *vs* C2DE groups for either campaign. For the bowel campaign, there was a significantly greater increase in awareness of blood in stools in the age group 75+ years compared with the 55–74 age group, and of looser stools in men compared with women. *Prompted symptom awareness:* Endorsement of ‘blood in poo' remained stable, overall and across different population subgroups. Men showed a significantly greater increase in endorsement of ‘looser poo' as a definite warning sign of bowel cancer than women. There were no significant differences across subgroups in endorsement of a 3-week cough as a definite warning sign of lung cancer. *GP attendances:* Overall, there were significant increases in attendances for symptoms directly linked to the campaigns, with the largest percentage increase seen in the 50–59 age group. For the bowel campaign, the increase was significantly greater for men and for practices in the most-deprived quintile, whereas for lung the increase was significantly greater for practices in the least-deprived quintile.

**Conclusions:**

The national bowel and lung campaigns reached their target audience and have also influenced younger and more affluent groups. Differences in impact within the target audience were also seen. There would seem to be no unduly concerning widening in inequalities, but further analyses of the equality of impact across population subgroups is warranted.

Reducing the ‘patient interval' (Weller *et al*, 2012) by encouraging prompt presentation after the onset of symptoms, which could be owing to cancer, has been a key part of the National Awareness and Early Diagnosis Initiative since its inception (Richards 2009). Evidence highlights that not realising the seriousness or significance of symptoms is a key determinant of the timeliness of help-seeking (Macleod *et al*, 2009; Forbes *et al*, 2014). This evidence, coupled with the often poor awareness of key cancer signs and symptoms, particularly in men and individuals from lower socioeconomic groups (Robb *et al*, 2009), provided the basis for the development of the Be Clear on Cancer (BCOC) programme of awareness campaigns.

The programme was initially led by the Department of Health (DH) in England and more latterly involves a partnership between DH, Public Health England and National Health Service England. It aims to provide members of the public with clear and concise information, highlighting the importance of symptoms and when to act. The programme represents, to our knowledge, the greatest national investment in cancer awareness and early diagnosis seen anywhere in the world. There is, therefore, large potential to expand the evidence base in this area through evaluation of this programme (Austoker *et al*, 2009).

The creative development process was led by advertising agency M&C Saatchi (London, UK). All campaigns within the BCOC programme have a consistent ‘look and feel' with coherent use of colour across all executions (posters, ads and leaflets) within a certain campaign (Be Clear on Cancer, 2014). The development process involved initial testing with the target audience to ensure relevance and appeal, and expert groups were convened to inform the development of the key campaign messages. GPs feature heavily in the creative design for most campaigns in an attempt to tackle head-on concerns about wasting the doctor's time, which are often highlighted as barriers to help-seeking (Robb *et al*, 2009; Forbes *et al*, 2013).

The campaigns aim to increase awareness of key cancer symptoms and to encourage help-seeking. For most of the campaigns, the advertising has been aimed at men and women from lower socioeconomic groups aged 50 years or over (55+ years for the early bowel activity) and their key influencers, such as friends and family. The use of media varies according to the level of roll-out, with TV featuring in regional and national activity. Sites for posters and channels and timings for radio and TV advertising are chosen to support the tailoring of the campaign towards the target audience.

Evaluation of the BCOC campaigns attempts to assess impact across the entire pathway, from awareness of symptoms, to presentations to GPs, through to urgent referrals for suspected cancer, diagnostic and treatment activity, stage of disease at diagnosis and, ultimately, survival and mortality data.

Given the potential for public health activity to, at least temporarily, exacerbate rather than reduce inequalities, particularly when the outputs can be viewed across the population (Lorenc *et al*, 2013), it is vital to understand the impact of the campaigns across demographic subgroups.

This paper focuses on two BCOC campaigns: the first national bowel campaign (from 30 January 2012 to 31 March 2012) and the first national lung campaign (from 8 May 2012 to 30 June 2012). These campaigns were based around the following key messages: ‘If you've had blood in your poo or looser poo for 3 weeks, your doctor wants to know' (bowel) and ‘If you've been coughing for 3 weeks or more, tell your doctor' (lung). Examples of the campaign posters are in the Supplementary Online Material. An overall evaluation of the impact of the lung campaign has previously been reported (Ironmonger *et al*, 2015).

This paper aims to investigate the impact on public awareness and the number of patients presenting to general practitioner (GP) practices with symptoms highlighted in the campaigns on samples of the population subgrouped by gender, age and a measure of socioeconomic status (SES).

## Materials and methods

The time periods over which data are collected, the samples included and the granularity at which they are available varies across all the BCOC evaluation metrics and are determined by a range of factors, including who has responsibility for providing the data and the basis upon which this is undertaken. With no routinely collected data available for the awareness and GP attendance measures, bespoke data collection was required. TNS BMRB (a market research company; London, UK) and Mayden (health care IT specialist) were commissioned to collect these data.

Using the data provided, overall our approach was to compare estimates of the measures of interest before and during or after the intervention for the sample as a whole and stratified by population subgroups.

### Public awareness

TNS BMRB undertook in-home face-to-face surveys with samples of respondents aged 55 years and over from across England. The choice of 55 years and over reflected the standard media-buying age boundary. Participants were chosen using a random location quota sampling technique (Crouch and Housden, 2003), and results were weighted to be representative of the population. Pre- and post-campaign interviews were conducted just before, and immediately after, the campaigns had run and involved different samples of the population. The surveys were informed by the Cancer Awareness Measure (Stubbings *et al*, 2009) and the bowel- (Power *et al*, 2011) and lung-specific versions (Simon *et al*, 2012). They covered a range of topics, but the results presented in this paper focus specifically on the responses to questions that captured respondents' awareness (unprompted and prompted) of bowel and lung cancer symptoms and, after being shown a clip of campaign adverts during the post-campaign survey interview, their views on whether the advertising had told them something new and whether they considered it relevant to them. See Supplementary Online Material for further details of data collection.

To determine whether there were differences in responses before and after the campaign, pre- and post-campaign survey responses were compared for the respondents overall and for population subgroups. For this, respondents were grouped by gender and age as 55–74 and 75+-year olds and, for an indicator of SES, each respondent was allocated to ‘ABC1' or ‘C2DE' based on their responses to a range of profiling questions (with ABC1 reflecting higher SES, and C2DE reflecting lower SES groups). Supplementary Online Material Table A provides the number of survey respondents by population subgroups. Changes in proportions in the survey results were tested for significance using a two-sample test of proportions. Differences in the magnitude of change between population subgroups (e.g., men *vs* women), proportional to the respective baseline levels of awareness, were tested for significance using Poisson regression models.

### GP attendances

To determine whether the number of patients presenting to GPs with key campaign symptoms increased after the launch of the campaigns, Mayden collected Read-code data (used by clinicians in electronic records) from a convenience sample of GP practices (*n*=355 for bowel, and *n*=486 for lung). Practices were recruited via the former Cancer Networks.

Participating practices were asked to supply data extracts of numbers of patients presenting with Read codes associated with symptoms directly linked to the campaign and a set of control symptoms over a specified time period (see Supplementary Online Material for more detail). Numbers of attendances with these symptoms during the campaign weeks were compared with numbers in the same time in the previous year, after adjustment for a 5-day week excluding public holidays (‘working days'). Comparisons were made for the sample overall and stratified by gender, age group (which was collected in 10-year age bands) and deprivation based on the Index of Multiple Deprivation 2010 for the Lower Super Output Area (LSOA) of the practices. Practices were allocated to quintiles of deprivation for LSOAs in England (Public Health England, 2013).

Changes in the number of attendances were tested using the *χ*^2^-test of two counts (Armitage *et al*, 2002). Differences in the magnitude of the change between population subgroups were tested using Poisson regression models. These tests assumed that there was no change in the size of the GP-registered populations.

*P*-values ⩽0.05 were considered to be statistically significant throughout the analyses.

## Results

### Public awareness

#### Unprompted awareness of cancer signs and symptoms

Respondents were asked to list as many warning signs and symptoms of bowel or lung cancer as possible (‘unprompted awareness'), during the respective campaign surveys. Overall, for the bowel campaign mentions of blood in stools significantly increased from 27% pre campaign to 42% post campaign (*P*<0.001) and mentions of looser stools increased from 10% to 23% (*P*<0.001). For the lung campaign, mentions of cough/hoarseness increased from 41% pre campaign to 50% post campaign (*P*=0.001; Table 1).

For the bowel campaign, the baseline (before campaign) level of unprompted awareness of blood in stools was similar for men and women (27% *vs* 27% *P*=0.957), but women had a higher baseline awareness of looser stools (6% for men *vs* 13% for women; *P*<0.001). For the lung campaign, baseline awareness was lower in men than in women (38% *vs* 44% *P*=0.024). There were pre to post increases in awareness for both men and women, with no significant differences in the magnitude of the shift between the genders for unprompted awareness of blood in stools (*P*=0.871) and cough/hoarseness (*P*=0.734), although post-lung campaign awareness of cough/hoarseness remained lower in men than in women (45% *vs* 55% *P*=0.001). Men saw a greater proportional increase in unprompted awareness of looser stools than women (from 6% to 18% for men *vs* 13% to 26% for women; *P*=0.038 for the difference). However, post-campaign levels remained lower for men than women (*P*=0.002).

There was no difference between the age groups for the baseline level of unprompted awareness of looser stools (10% for 55–74 years *vs* 8% for 75+ years; *P*=0.159), but for unprompted awareness of blood in stools and cough/hoarseness respondents aged 75+ had a lower baseline level of awareness than those aged 55–74 years (15% *vs* 31% (*P*<0.001), respectively, for bowel; 35% *vs* 43% (*P*=0.016) for lung). There was a significantly greater increase in awareness of blood in stools pre to post campaign for respondents in the 75+ age group than in the 55–74 age group (31–45% for 55–74 years and 15–33% for 75+ years; *P*=0.031 for the difference), but the post-campaign level for the older group was still lower than that of the younger group (*P*=0.001). There was no difference in the magnitude of change in awareness of looser stools between the age groups (*P*=0.495); however, the post-campaign levels of awareness were higher in the 55–74 age group than in the 75+-year age group (*P*=0.001). The results for the lung campaign were somewhat different, with no significant change in pre- and post-campaign results for the 75+ age group (*P*=0.721), compared with an 11 percentage point increase for the 55–74 age group (*P*<0.001); however, the magnitude of change was not significantly different between age groups (*P*=0.226).

For both campaigns, ABC1 and C2DE respondents saw significant increases in unprompted awareness from from pre to post campaign, with no significant differences in the magnitude of shift between the groups (*P*=0.424 for blood in stools, *P*=0.163 for looser stools and *P*=0.944 for cough/hoarseness). Baseline levels of awareness were lower in C2DEs than in ABC1s, and they remained lower in the post-campaign surveys for awareness of blood in stools (37% *vs* 48%, *P*<0.001) and cough/hoarseness (45% *vs* 56% *P*=0.001). There was no difference in the post-campaign levels of awareness of looser stools (25% for ABC1s *vs* 21% for C2DEs; *P*=0.224).

#### Prompted awareness of cancer signs and symptoms

Respondents were asked to indicate the extent to which they thought that the listed symptoms were a warning sign of bowel or lung cancer (‘prompted awareness'). For the bowel campaign, overall the proportion saying ‘blood in your poo for 3 weeks or longer' was a definite warning sign of bowel cancer remained at around the same level before and after campaign (55–56% *P*=0.751), and there were no significant changes within any of the population subgroups (Table 2). However, there was an overall increase in prompted awareness of ‘looser poo for 3 weeks or longer' as a definite warning sign of bowel cancer (from 17% before to 27% after; *P*<0.001). Baseline awareness was lower in men than in women (13% *vs* 22% *P*<0.001), but there was a significantly greater increase in the proportion of men endorsing ‘looser poo' as a definite warning sign compared with women (13–26% for men *vs* 22–28% for women; *P*=0.013 for the difference), bringing post-campaign awareness to similar levels (*P*=0.456). Although significant increases were seen in prompted awareness of ‘looser poo' for the age and socioeconomic groups, there were no significant differences in the magnitude of the changes between the 55–74 and 75+ age groups (*P*=0.643) or between the ABC1 and C2DE (*P*=0.279) groups.

For the lung campaign, the overall proportion saying a ‘cough for 3 weeks or more that doesn't go away' was a definite warning sign of lung cancer rose from 18% to 33% pre to post campaign (*P*<0.001). This prompted awareness was similar between men and women in the baseline survey (17% *vs* 19% *P*=0.389), and significant increases were seen for both men and women pre to post campaign, with no significant difference in the magnitude of change between genders (17–33% for men *vs* 19–33% for women; *P*=0.587 for the difference). Similar patterns were seen for the age and socioeconomic breakdowns.

#### Campaign relevance and ‘new news'

After answering the symptom awareness and other questions, respondents were shown a clip of the campaign advert and asked the extent to which they agreed that the campaign told them something new and was relevant to them. Overall, around half of the survey respondents thought that the advertising told them something new (51% bowel and 46% lung) and more than half of the survey respondents thought that the advertising was relevant to them (67% bowel and 55% lung; Table 3). Men were more likely to think that the advertising told them something new for both campaigns (55% *vs* 48% *P*=0.020 for bowel; 50% *vs* 43% *P*=0.022 for lung). There was also a trend for men to perceive the advertising as more relevant, although this was not statistically significant for the lung campaign (70% *vs* 64% *P*=0.047 for bowel; 57% *vs* 53% *P*=0.249 for lung).

By age group, those aged 55–74 years were more likely to consider the adverts from both campaigns to be telling them something new and to be relevant to them than the 75+ age group (new news: for bowel 53% *vs* 44% *P*=0.014; for lung 49% *vs* 38% *P*=0.002. Advertising relevant: for bowel 70% *vs* 57% *P*<0.001; for lung 57% *vs* 48% *P*=0.008). By socioeconomic group, there was a trend for C2DE respondents to more often agree that the adverts told them something new (for bowel 47% for ABC1s *vs* 54% for C2Des; *P*=0.037; for lung 43% *vs* 49% *P*=0.040). In terms of agreeing that the adverts were relevant, there was no significant difference between ABC1 and C2DE groups following the bowel campaign (*P*=0.439), but C2DE respondents were significantly more likely than ABC1s to think that the lung advert was relevant to them (50% *vs* 58% *P*=0.009).

### GP attendance

On the basis of presentations of patients aged 50+ years, there were significant increases in attendances for symptoms directly related to the advertising for both campaigns during the campaign weeks in 2012 compared with the same weeks in 2011 (29% and 63% for the bowel and lung campaigns, respectively; Table 4), which were significantly greater than the changes for visits with control symptoms (−6% and 2%, respectively; Supplementary Online Material Table B). For both campaigns, there were significant increases in attendances for both men and women. For the bowel campaign, the percentage increase in GP visits for men was significantly greater than the increase for women (37% *vs* 22% *P*=0.004), with no gender difference in the change in attendances for control symptoms. For the lung campaign, there was no difference in the magnitude of the increase between the two genders (66% for men *vs* 61% for women; *P*=0.107), whereas there was a small significant gender difference in the change in attendances for control symptoms (−1% change for men *vs* 5% for women; *P*=0.001).

All age groups, across both campaigns, saw a significant increase in visits for campaign-related symptoms from 2011 to 2012, with the exception of the under-20s age group for bowel symptoms. For both the bowel and lung campaigns, the largest percentage increase was seen in the 50–59-year age group (54%, *P*<0.001 for bowel; 88%, *P*<0.001 for lung). The lung campaign saw a more sizeable increase in the under-50s age group than in the 50+ age group (70% *vs* 63%, respectively; *P*=0.001), whereas there was no significant difference between the under-50s and 50+ age groups in the bowel campaign (28% *vs* 29% *P*=734).

For comparison, visits for control symptoms decreased more for those aged 50+ than those aged <50 years for the bowel campaign (a non-significant −0.1% decrease for <50 years and −6% for 50+ years; *P*=0.002 for the difference). For the lung campaign, a greater increase in visits was seen in the under-50 age group (7% for <50 years and 2% for 50+ years; *P*=0.003). For both campaigns, the largest increase in visits for control symptoms was seen in the under-20s age group (15% for bowel and 12% for lung).

On the basis of the location of the GP practice, the highest percent change in attendances for campaign symptoms for patients aged 50 years or more was for practices in the most-deprived quintile (the first quintile) for the bowel campaign (72% increase) and for practices in the fourth quintile for the lung campaign (78% increase). There was a significantly greater increase in attendances for patients aged 50 years or more for bowel campaign symptoms in practices in the most-deprived quintile compared with practices in the least-deprived quintile (72% *vs* 18% *P*<0.001). For the lung campaign, the opposite pattern was seen, with a significantly greater increase in attendances for campaign-related symptoms for practices in the least-deprived quintile compared with those in the most-deprived quintile (67% *vs* 48% *P*=0.003).

For both the bowel and lung campaigns, there was no difference in the magnitude of change for GP attendances with control symptoms between practices in the most- and least-deprived quintiles (*P*=0.892 bowel and *P*=0.768 lung).

## Discussion

The BCOC campaigns for bowel and lung cancer were aimed at men and women aged 50 years and over from lower socioeconomic groups. The results suggest that the campaigns reached the broad target audience, but also reached younger and more affluent audiences as well. This is likely to reflect the use of channels such as television and radio where tailoring to an audience is possible, but only to a degree.

Understanding the variation in impact within the target audience is essential and can be used to inform future campaign development and implementation.

### Bowel campaign

Results from the national bowel campaign evaluation suggest that the campaign was seen as relevant across the socioeconomic spectrum. Although those aged 55–74 years were more likely to agree that the campaign told them something new and was relevant, the increase in unprompted awareness suggests that the awareness messages reached the older subgroup of the target audience (75+ years). The campaign seemed to have resonated particularly with men, who saw a greater increase in unprompted awareness of looser stools than women, were more likely to endorse ‘loose poo' as a definite warning sign of bowel cancer and agree that the campaign had told them something new. There was also a tendency for men to more often consider the campaign as relevant to them. This may have been behind the greater increase in GP visits for men compared with women. Encouragingly, there was a significantly larger increase in GP attendances for practices in the most-deprived quintile compared with the least-deprived quintile. Interestingly, the numbers of GP visits and increases seen were not of the same magnitude as those seen with the lung campaign. It would be useful to know whether this reflects prevalence of bowel symptoms within the community or whether there is something else at play.

### Lung campaign

In terms of awareness, the magnitude of increase in unprompted awareness was similar in men and women, but men still had lower levels after campaign. There were no significant differences in endorsement of a 3-week cough as a definite warning sign of lung cancer across the different population subgroups. As was seen in the bowel campaign, the 55–74 age group was more likely to agree that the lung campaign provided new news and was relevant, and unprompted awareness results suggest that the campaign reached the 55–74 age group but not the 75+ age group. C2DE respondents were more likely to consider the campaign relevant than those in the ABC1 group, which is encouraging given the known links between smoking, lung cancer and deprivation (National Cancer Intelligence Network, 2014a). However, the data suggest a greater increase in GP attendances for practices in less-deprived areas.

### Implications

Overall, the results seen for men are encouraging. A number of the socioeconomic breakdown results are also encouraging, and on the whole they suggest no unduly concerning widening between groups. However, the greater increase in GP attendances for practices in the least-deprived quintile compared with the most-deprived quintile for the lung campaign warrants consideration, and further efforts are needed to increase engagement with, and impact in, lower socioeconomic groups. The age breakdown data suggest that the older age groups may not be receiving and responding to the campaigns in the same way as the younger age groups, even within the broad target audience of those aged 50 and over. This is important given that advancing age is associated with poorer cancer outcomes including cancer survival (De Angelis *et al*, 2014; National Cancer Intelligence Network, 2014b) and warrants further attention.

### Limitations

The data on which these analyses are based stem from different sources, and this is associated with variation in samples, sociodemographic information and time periods covered. Furthermore, each source is associated with its own limitations; the use of Read-code data for GP attendances, for example, is reliant on accurate and consistent coding by GPs, and thus there may be an element of underestimation, as well as potential misrepresentation, given the convenience nature of the sample. This, coupled with the observational design and cross-sectional nature of the metrics, affects the extent to which the results presented can be definitively linked to the campaign activity. However, the approach to data collection and analysis gives some assurances that the results do reflect campaign activity, at least in part. How the effects are sustained after the campaign activity has been completed is an important area, and although there is some evidence that impact on awareness may be sustained several months after a campaign has ended, at least for the lung campaign (Power and Wardle, 2015), this is the focus of other analyses. It is hoped that this initial analysis will encourage others to conduct further work in this area, reinforce the importance of considering the impact of the campaigns both within and outside the target audience and stimulate improvements in the collection and consistency of information which allow sociodemographic comparisons, such as in the case of ethnicity.

## Conclusion

Reducing the ‘patient interval' is an important part of the strategy to reduce late diagnosis of cancer and to improve cancer outcomes. These initial analyses of the first national Be Clear on Cancer (BCOC) bowel and lung campaign results support that the campaigns are reaching their broad target audiences, but also younger and more affluent audiences. There would seem to be no unduly concerning widening in inequalities, and in some instances it looks like the campaigns may have had an impact on reducing gaps between groups. For some areas, however, there is still a way to go to bring all groups to the level of the highest. Additional exploration of this is warranted, and further understanding the impact of the campaigns across different groups should be used to inform the future development and implementation of the programme, as well as other approaches to encouraging early presentation of symptoms.

## Figures and Tables

**Table 1:** Pre- and post-campaign unprompted awareness of bowel and lung cancer symptoms mentioned in the campaign adverts. Proportion of respondents mentioning specific symptoms, overall and broken down by population subgroups.

	**Population subgroup**	**Pre-campaign % (no.)**	**Post-campaign % (no.)**	**Percentage point change**^a^ **(95% CI)**	***P*****-value for change pre- to post-campaign**	***P*****-value for between-group difference**^b^
**Bowel campaign**						
Unprompted awareness of blood in stools	Overall	27 (314)	42 (449)	14 (10 to 18)	<0.001	–
	Men	27 (151)	41 (199)	14 (8 to 20)	<0.001	0.871
	Women	27 (163)	42 (251)	15 (9 to 20)	<0.001	
	55–74 years	31 (271)	45 (361)	13 (9 to 18)	<0.001	0.031
	75+ years	15 (42)	33 (89)	18 (11 to 25)	<0.001	
	ABC1	33 (177)	48 (226)	15 (9 to 21)	<0.001	0.424
	C2DE	23 (137)	37 (224)	15 (9 to 20)	<0.001	
Unprompted awareness of looser stools	Overall	10 (111)	23 (245)	13 (10 to 16)	<0.001	–
	Men	6 (31)	18 (89)	13 (9 to 17)	<0.001	0.038
	Women	13 (80)	26 (156)	13 (8 to 17)	<0.001	
	55–74 years	10 (90)	25 (205)	15 (11 to 19)	<0.001	0.495
	75+ years	8 (21)	15 (40)	7 (2 to 13)	0.006	
	ABC1	12 (65)	25 (116)	12 (8 to 17)	<0.001	0.163
	C2DE	8 (46)	21 (129)	14 (10 to 18)	<0.001	
**Lung campaign**						
Unprompted awareness of cough/hoarseness	Overall	41 (478)	50 (560)	8 (4 to 13)	<0.001	–
	Men	38 (203)	45 (228)	7 (1 to 13)	0.030	0.734
	Women	44 (275)	55 (332)	10 (4 to 16)	<0.001	
	55–74 years	43 (378)	55 (453)	11 (6 to 16)	<0.001	0.226
	75+ years	35 (100)	37 (107)	1 (−6 to 9)	0.721	
	ABC1	46 (242)	56 (277)	9 (3 to 15)	0.003	0.944
	C2DE	37 (236)	45 (283)	8 (3 to 13)	0.004	

Abbreviation: CI=confidence interval.

a Percentage point changes may not directly equal post-campaign minus pre-campaign values owing to rounding.

b For the test of the between-group difference in the magnitude of change pre to post campaign.

**Table 2:** Pre- and post-campaign prompted awareness of bowel and lung symptoms mentioned in the campaign adverts. Respondents recognising symptoms as definite warning signs of cancer, overall and by population subgroups.

	**Population subgroup**	**Pre-campaign % (no.)**	**Post-campaign % (no.)**	**Percentage point change**^a^ **(95% CI)**	***P*****-value for change pre- to post-campaign**	***P*****-value for between-group difference**^b^
**Bowel campaign**						
Prompted awareness of ‘blood in your poo for 3 weeks or longer'	Overall	55 (634)	56 (603)	1 (−3 to 5)	0.751	—
	Men	52 (288)	55 (266)	3 (−3 to 9)	0.367	0.492
	Women	58 (346)	57 (337)	−2 (−7 to 4)	0.597	
	55–74 years	57 (490)	57 (461)	0 (−4 to 5)	0.868	0.907
	75+ years	52 (144)	53 (141)	1 (−7 to 10)	0.780	
	ABC1	57 (309)	54 (256)	−3 (−9 to 3)	0.322	0.277
	C2DE	54 (325)	58 (347)	4 (−2 to 9)	0.181	
Prompted awareness of ‘poo that is looser than usual for 3 weeks or longer'	Overall	17 (200)	27 (295)	10 (6 to 13)	<0.001	—
	Men	13 (70)	26 (127)	14 (9 to 18)	<0.001	0.013
	Women	22 (130)	28 (168)	6 (1 to 11)	0.011	
	55–74 years	17 (151)	28 (226)	10 (7 to 14)	<0.001	0.643
	75+ years	18 (49)	25 (69)	8 (1 to 15)	0.024	
	ABC1	18 (96)	25 (118)	7 (2 to 12)	0.005	0.279
	C2DE	17 (103)	29 (176)	12 (7 to 17)	<0.001	
**Lung campaign**						
Prompted awareness of cough for 3 weeks or more that doesn't go away'	Overall	18 (206)	33 (373)	15 (12 to 19)	<0.001	—
	Men	17 (90)	33 (169)	16 (11 to 21)	<0.001	0.587
	Women	19 (116)	33 (204)	15 (10 to 20)	<0.001	
	55–74 years	17 (150)	34 (284)	17 (13 to 21)	<0.001	0.175
	75+ years	20 (57)	31 (89)	10 (3 to 18)	0.004	
	ABC1	17 (90)	32 (161)	15 (10 to 20)	<0.001	0.925
	C2DE	18 (116)	34 (212)	16 (11 to 20)	<0.001	

Abbreviation: CI=confidence interval.

a Percentage point changes may not directly equal post-campaign minus pre-campaign values owing to rounding.

b For the test of the between-group difference in the magnitude of change before to after campaign.

**Table 3:** Proportion of respondents agreeing that the advertising told them something new and is relevant to them, overall and by population subgroups for the bowel and lung campaigns (results from the post-campaign survey)

	**Bowel campaign**			**Lung campaign**		
**Population subgroup**	**% (No.)**	**Percent point difference between subgroups (95% CI)**	***P*****-value for difference between subgroups**	**% (No.)**	**Percent point difference between subgroups (95% CI)**	***P*****-value for difference between subgroups**
**Net agreement**^a^ **that advertising told something new**						
Overall	51 (546)	—	—	46 (521)	—	—
Men	55 (264)	−7 (−13 to −1)	0.020	50 (257)	−7 (−13 to −1)	0.022
Women	48 (282)			43 (264)		
55–74 years	53 (428)	−9 (−16 to −2)	0.014	49 (409)	−11 (−17 to −4)	0.002
75+ years	44 (118)			38 (112)		
ABC1	47 (223)	6 (0 to 12)	0.037	43 (214)	6 (0 to 12)	0.040
C2DE	54 (323)			49 (306)		
**Net agreement**^a^ **that advertising was relevant**						
Overall	67 (719)	—	—	55 (614)	—	—
Men	70 (338)	−6 (−11 to 0)	0.047	57 (290)	−3 (−9 to 2)	0.249
Women	64 (381)			53 (324)		
55–74 years	70 (568)	−14 (−20 to −7)	<0.001	57 (474)	−9 (−16 to −2)	0.008
75+ years	57 (151)			48 (140)		
ABC1	68 (322)	−2 (−8 to 3)	0.439	50 (251)	8 (2 to 14)	0.009
C2DE	66 (397)			58 (363)		

Abbreviation: CI=confidence interval.

a All patients who strongly or slightly agreed.

**Table 4:** Number of presentations to GP practices with the symptoms related to the bowel and lung campaign advertising, during the respective campaign weeks in 2012 compared with the same weeks in 2011, adjusted for working days. For practices in the sample: *n*=355 bowel and *n*=486 lung.

		**Number of GP attendances**				
	**Population subgroup**	**2011**	**2012**	**Percent change (95% CI)**	***P*****-value for percent change**	***P*****-value for between-group difference**^b^
**Bowel campaign (attendances of patients with blood in stools/looser stools-related symptoms)**						
Gender^a^	Men	1947	2673	37 (30 to 44)	<0.001	0.004
	Women	2208	2692	22 (16 to 28)	<0.001	
Age (years)	Under 20s	669	647	−3 (−14 to 7)	0.544	—
	20–29	644	815	27 (15 to 38)	<0.001	
	30–39	699	923	32 (21 to 43)	<0.001	
	40–49	942	1392	48 (38 to 58)	<0.001	
	50–59	1049	1620	54 (45 to 64)	<0.001	
	60–69	1157	1487	29 (20 to 37)	<0.001	
	70–79	1100	1275	16 (7 to 25)	<0.001	
	80+	853	994	17 (7 to 26)	0.001	
	Under 50s	2954	3777	28 (22 to 33)	<0.001	0.734
	50+	4159	5376	29 (25 to 34)	<0.001	
Deprived quintile^a^	1 (most deprived)	246	422	72 (51 to 92)	<0.001	<0.001^c^
	2	914	1185	30 (20 to 39)	<0.001	
	3	1292	1797	39 (31 to 48)	<0.001	
	4	1329	1527	15 (7 to 23)	<0.001	
	5 (least deprived)	361	425	18 (3 to 33)	0.022	
**LUNG campaign (attendances of patients with cough-related symptoms)**						
Gender^a^	Men	8002	13 250	66 (62 to 69)	<0.001	0.107
	Women	10 392	16 692	61 (58 to 64)	<0.001	
Age (years)	Under 20s	8755	14 298	63 (60 to 67)	<0.001	—
	20–29	1883	3404	81 (73 to 88)	<0.001	
	30–39	2447	4324	77 (70 to 83)	<0.001	
	40–49	3648	6499	78 (73 to 84)	<0.001	
	50–59	4091	7675	88 (82 to 93)	<0.001	
	60–69	5632	9504	69 (64 to 73)	<0.001	
	70–79	5025	7664	53 (48 to 57)	<0.001	
	80+	3742	5291	41 (36 to 46)	<0.001	
	Under 50s	16 733	28 524	70 (68 to 73)	<0.001	0.001
	50+	18 490	30 133	63 (61 to 65)	<0.001	
Deprived quintile^a^	1 (most deprived)	1736	2561	48 (40 to 55)	<0.001	0.003^c^
	2	4568	7368	61 (57 to 66)	<0.001	
	3	5187	7989	54 (50 to 58)	<0.001	
	4	4899	8704	78 (73 to 82)	<0.001	
	5 (least deprived)	2100	3511	67 (60 to 74)	<0.001	

Abbreviations: CI=confidence interval; GP=general practitioner.

a Patients aged 50+ years.

b For the test of the between-group difference in the magnitude of change pre to post campaign.

c *P*-value for difference in percentage change between most- and least-deprived quintiles only.

## Notes

Cancer Research UK has been involved in the development and leadership of the National Awareness and Early Diagnosis Initiative, which includes the Be Clear on Cancer programme among other activities. Cancer Research UK was funded by the DH to coordinate the evaluation of Be Clear on Cancer campaigns running until the end of March 2013. GR co-authored the evaluation report on the regional BCOC bowel campaign. SD is national clinical director for cancer and is involved in the BCOC programme on an ongoing basis.
