# Supplementary material for: The impact of national cancer awareness campaigns for bowel and lung cancer symptoms on sociodemographic inequalities in immediate key symptom awareness and GP attendances
Source: Br J Cancer. 2015 Mar 3;112(Suppl 1):S14–21. doi: 10.1038/bjc.2015.31 (PMC4385971; doi:10.1038/bjc.2015.31)
Supplement: Supplementary Materials and Methods [file bjc201531x1.docx]

**Supplementary Materials and Methods**

**Campaign materials**

Figure 1. Example of the national bowel campaign poster


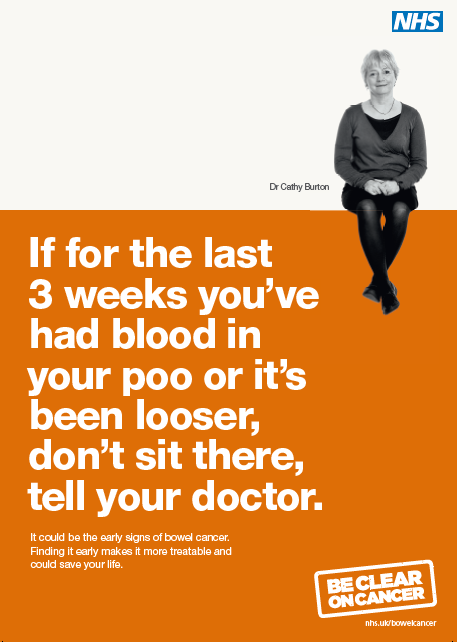


Figure 2. Example of the national lung campaign poster
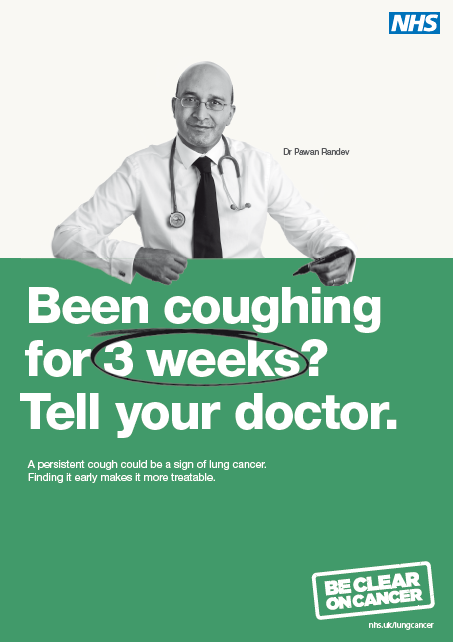


**Methods**

1. Public awareness

The overall purpose of the pre- and post-campaign surveys carried out by TNS-BMRB (2014) was to evaluate the campaign and assess campaign performance in terms of: awareness of signs and symptoms of cancer; recognition of campaign messages; beliefs and attitudes towards cancer and early diagnosis; self-efficacy related to visiting the GP; and emotional engagement with the advertising.

The questions were added to the TNS omnibus survey which was carried out across England. The interviews took 15 minutes in the pre-campaign survey and 20 minutes in the post- survey and were conducting by trained interviewers using in-home computer assisted personal interviewing.

**Random location quota sampling:**

The sampling technique used in this survey was a tightly controlled form of random location quota sampling developed within legacy BMRB (and it is the basis of most consumer surveys which TNS-BMRB conducts).

The aim of random location sampling is to eliminate the more unsatisfactory features of quota sampling without incurring the cost and other penalties involved in conducting surveys according to strict probability methods.

The principal distinguishing characteristic of random location quota sampling, as operated by TNS BMRB, is that interviewers are given very little choice in the selection of respondents. Respondents are drawn from a small set of homogenous streets, selected with probability proportional to population after stratification by their Acorn characteristics (CACI, 2014) and area. Quotas are set in terms of characteristics which are known to have a bearing on individuals' probabilities of being at home and so available for interview. Rules are given which govern the distribution, spacing and timing of interviews.

For the omnibus survey, the sample of areas takes as its universe all sample units (groups of Census 2001 Output Areas, on average, 300 households) in Great Britain. Output areas are stratified in the following manner:

1. Standard Area
2. Within Standard Area - by Acorn type
3. Within Standard Area by County and ITV Area

Thus, the design is single stage, using direct selection of appropriate groups of output areas, rather than taking streets at random from larger units such as wards or parishes.

Quotas were set by sex (male, female housewife, female non-housewife); within female housewives, presence of children and working status, and within men, working status in order to ensure a balanced sample of adults within effective contacted addresses. Interviewing assignments were conducted over two days of fieldwork and carried out on weekdays from 2pm–8pm and at the weekend. Interviewers were instructed to leave three doors between each successful interview.

**Weighting:**

Data were weighted to be representative of the population: a single cell matrix was used in which the matrix consisted of age (55-64 and 65+) by gender (male and female) by Government Office Region (GOR) using the nine GORs in England. Targets were taken from the BARB (Broadcasters’ Audience Research Board) Establishment Survey 2 Years Ending December 2008.

Survey questions looked at in this paper (questions from bowel campaign survey are used as an example):

| **UNPROMPTED AWARENESS:**  **There are many signs and symptoms of bowel cancer. Please write in as many as you are aware of.**    **PROMPTED AWARENESS:**  **I’m going to list some symptoms that may or may not be warning signs for bowel cancer. Please be reassured that having one of these signs or symptoms does not necessarily mean that you have bowel cancer but simply that it should be investigated further. For each one can you tell me the extent to which you think it is a warning sign for bowel cancer?**  *[Options: Is definitely not a warning sign/Is probably not a warning sign/Is probably a warning sign/Is* *definitely* *a warning sign/Don’t know/Refused]*   - Bleeding from your back passage for three weeks or longer - A pain or lump in your tummy - Poo that is looser than usual, for three weeks or longer - A feeling that your bowel does not completely empty after using the toilet - Blood in your poo for three weeks or longer - Losing weight for no obvious reason - Going to the toilet for a poo more frequently, for three weeks or longer - Feeling more tired than usual for some time   **VIEWS ON CAMPAIGN ADVERTISING:**  **I am now going to read out some statements about the advertising you have seen and heard. Thinking about all of this advertising, please tell me to what extent you agree or disagree with each statement.** *[Options: Agree strongly/Agree slightly/Neither agree nor disagree/Disagree slightly/Disagree strongly/Don’t know]*   - The advertising is relevant to you - The advertising told you something new - This advertising stands out from other advertising - This advertising is clear and easy to understand - It is important that adverts like this are shown - This advertising would make you more likely to go to your GP or doctor if you had any of these symptoms and felt concerned about them |
| --- |

Throughout the surveys, responses to open-ended questions (including unprompted awareness of bowel/lung cancer symptoms) were coded and allocated to a number of categories.

**Allocation to social grade (ABC1 vs. C2DE):**

Social grade was determined in accordance with Market Research Society (2014) guidelines, primarily based on the occupation of the Chief Income Earner in the household.

Respondents were asked the following questions:

| - **Which member of your household is the Chief Income Earner (CIE) - that is the person with the largest income whether from employment, pensions, state benefits, investments or any other sources?** *[Respondent, Respondent’s spouse/partner, Other]* - **Working status of CIE** *[Employed, Not employed, Not working - dependent of state benefit, Not working - other income]*   Collect occupation or previous occupation details of CIE:   - **What type is the type of firm where the CIE works?** *[open ended]* - **What is the type of job actually done by the CIE?** *[open ended]* - **What is the title, rank, grade etc of the CIE?** *[open ended]* - **How many is CIE responsible for?** *[open ended]* - **Does the CIE have any qualifications (such as apprenticeships, professional qualifications, university degrees, diplomas etc)?** *[Yes or No]* - **Enter qualifications** - **Enter any other relevant details to assist classification of occupation and industry** - **Enter address** |
| --- |

ABC1 refers to workers who are: higher or intermediate managerial, administrative and professional; and supervisory, clerical and junior managerial, administrative and professional.

C2DE refers to: skilled manual workers; semi-skilled & unskilled manual workers; and sate pensioners, casual and lowest grade workers and those that rely on welfare, including students (National Readership Survey, 2014).

1. GP attendances

Mayden (2014) invited GP practices to take part in this aspect of the evaluation through the (former) cancer network leads.

There was some overlap (82 practices) between the practices that contributed data for the lung and bowel campaign evaluations.

Lists of symptoms and their corresponding Read codes were arrived at with the support of primary care colleagues from the Department of Health Policy Research Unit in cancer awareness, screening and early diagnosis, with specific contributions from clinicians at the University of Durham and Peninsular Medical School supported by additional evidence from the Mount Vernon Cancer Network and the Greater Manchester and Cheshire Cancer Network.

The GP practice Index of Multiple Deprivation (IMD) 2010 score was estimated by taking a weighted average of the IMD scores for each LSOA (Lower Super Output Area) in which a given practice has registrations. The weights were the percent of the practice's registrations in each LSOA.

Bowel

For the bowel campaign, practices were required to be users of the EMIS LV system to participate. Practices were provided with a process document to follow to guide them through a ‘search’ for selecting data meeting the criteria and on how to present the information in Excel format. Data were extracted for 1^st^ December 2010 to 31^st^ May 2012.

**List of bowel campaign related symptom Read codes:**

| **Read code** | **Description** |
| --- | --- |
| **Rectal bleed** | |
| 19E4 | Black faeces |
| 19E4-12 | C/O - melaena |
| 19E6 | Blood in faeces |
| 196B | Painful rectal bleeding |
| 196C | Painless rectal bleeding |
| 19ED | Blood on toilet paper |
| J573.00 | Haemorrhage of rectum and anus |
| J573.11 | Bleeding PR |
| J5730.00 | Rectal haemorrhage |
| J5730.11 | Rectal bleeding |
| J5730.12 | PRB - Rectal bleeding |
| J5731 | Anal haemorrhage |
| J573z | Haemorrhage of rectum and anus NOS |
| **Change in bowel habit** | |
| 19EA | Change in bowel habit |
| 19EA.11 | Altered bowel habit |
| 19EE | Increased frequency of defaecation |
| 19EF | Urgent desire for stool |
| **Loose stools** | |
| 19F.12 | Loose stools |
| R0771 | [D] Stools loose |

The selected control symptoms were migraine, headache, depressive episode and depressed.

Lung

For the lung campaign, data from four practice systems were included: EMIS LV, EMIS Web, SystmOne and Vision. A process document was developed for each system for the practices to follow.

Two versions of Read code terms were used: version 2 for EMIS LV, EMIS Web and Vision users; and version 3 for SystmOne users (the two versions do not directly map on to each other). Data were extracted for 1^st^ March 2010 to 31^st^ May 2013.

**List of lung campaign related symptom Read codes:**

| **Read V2** | **Description** | **Read V3** | | **Description** |
| --- | --- | --- | --- | --- |
| 1712.00 | Dry cough | 171A. | Chronic cough | |
| 1713.00 | Productive cough -clear sputum | H3101 | Smokers' cough | |
| 1714.00 | Productive cough -green sputum | R062. | [D]Cough | |
| 1715.00 | Productive cough-yellow sputum | X76I8 | Dry cough | |
| 1716.11 | Coughing up phlegm | Xa2kc | Persistent cough | |
| 1716.00 | Productive cough NOS | XaFwR | Unexplained cough | |
| 1717.00 | Night cough present | XE0qn | Cough | |
| 1719.11 | Bronchial cough | XM0Ch | C/O - cough | |
| 1719.00 | Chesty cough | X76Hy | Productive cough | |
| 171..12 | Sputum - symptom | X76I3 | Sputum - symptom | |
| 171..11 | C/O - cough | X76IA | Producing sputum | |
| 171..00 | Cough | 1719. | Chesty cough | |
| 171A. | Chronic cough | 1719. | Bronchial cough | |
| 171B.00 | Persistent cough | 171C. | Morning cough | |
| 171C.00 | Morning cough | 171D. | Evening cough | |
| 171D.00 | Evening cough | Xa4fN | Barking cough | |
| 171E.00 | Unexplained cough | Xa7u8 | Observation of cough | |
| 171F.00 | Cough with fever | Xa7u9 | Brassy cough | |
| 171H.00 | Difficulty in coughing up sputum | Xa7uA | Bovine cough | |
| 171J.00 | Reflux cough | Xa7uB | Effective cough | |
| 171K.00 | Barking cough | Xa7uC | Cough reflex | |
| 171Z.00 | Cough symptom NOS | XaIO1 | Cough with fever | |
| 173B.00 | Nocturnal cough / wheeze | XaLCS | Reflux cough | |
| H3101 | Smokers' cough | XC07I | Coughing - function | |
| R062.00 | [D]Cough |  |  | |
| R0620 | [D]Cough syncope |  |  | |
| R0630 | [D]Cough with haemorrhage |  |  | |
| S120A00 | Cough fracture |  |  | |
| S127100 | Cough fracture of ribs |  |  | |

The selected control symptoms were urinary tract infection, neck pain, shoulder pain and knee pain

**References**

CACI (2014). What is Acorn? <http://acorn.caci.co.uk/> (Accessed Nov 2014).

Market Research Society (2014), Occupational Groupings <https://www.mrs.org.uk/intelligence/occupational_groupings> (Accessed Nov 2014).

Mayden (2014). Homepage <http://www.mayden.co.uk/> (Accessed Nov 2014).

National Readership Survey (2014). Lifestyle and classification data.

<http://www.nrs.co.uk/nrs-print/lifestyle-and-classification-data/> (Accessed Nov 2014).

TNS BMRB (2014). Homepage <http://www.tns-bmrb.co.uk/> (Accessed Nov 2014).
